# Phenotypic Skewing of Macrophages *In Vitro* by Secreted Factors from Colorectal Cancer Cells

**DOI:** 10.1371/journal.pone.0074982

**Published:** 2013-09-18

**Authors:** Sofia Edin, Maria L. Wikberg, Jörgen Rutegård, Per-Arne Oldenborg, Richard Palmqvist

**Affiliations:** 1 Department of Medical Biosciences, Pathology, Umeå University, Umeå, Sweden; 2 Department of Surgical and Perioperative Sciences, Surgery, Umeå University, Umeå, Sweden; 3 Department of Integrative Medical Biology, Section for Histology and Cell Biology, Umeå University, Umeå, Sweden; University of Bari & Consorzio Mario Negri Sud, Italy

## Abstract

Macrophages are cells with many important functions in both innate and adaptive immune responses and have been shown to play a complex role in tumor progression since they harbour both tumor preventing (M1 macrophages) and tumor promoting (M2 macrophages) activities. In many human cancers, infiltrating macrophages have been associated with a poor patient prognosis, and therefore suggested to be mainly of an M2 phenotype. However, we and others have previously shown that increased macrophage density in colorectal cancer (CRC) instead is correlated with an improved prognosis. It is an intriguing question if the different roles played by macrophages in various cancers could be explained by variations in the balance between M1 and M2 macrophage attributes, driven by tumor- or organ-specific factors in the tumor microenvironment of individual cancers. Here, we utilized an *in vitro* cell culture system of macrophage differentiation to compare differences and similarities in the phenotype (morphology, antigen-presentation, migration, endocytosis, and expression of cytokine and chemokine genes) between M1/M2 and tumor activated macrophages (TAMs), that could explain the positive role of macrophages in CRC. We found that secreted factors from CRC cells induced TAMs of a “mixed” M1/M2 phenotype, which in turn could contribute to a “good inflammatory response”. This suggests that re-education of macrophages might allow for important therapeutic advances in the treatment of human cancer.

## Introduction

The tumor microenvironment plays several complex and important roles in tumor progression [1]. During the past decade, one main focus in cancer research has been on the importance of the inflammatory tumor microenvironment, which has subsequently led to the inclusion of tumor-promoting inflammation and immune evasion as emerging hallmarks of cancer [2]. An increased understanding of the immune contexture - i.e. the location, density and functional orientation of immune cells, and how this affects tumor progression might provide important tools for the prediction of patient prognosis as well as the development of new treatment strategies [3].

The work on inflammation in human cancer has resulted in the identification of components of the immune system that can be both beneficial and deleterious for patient prognosis. One such component is the macrophages of the innate immune system. Macrophages are shown to be highly plastic cells that can display both tumor preventing (M1 macrophages) and tumor promoting functions (M2 macrophages) reviewed in [4], [5]. In brief, the classically activated M1 macrophages support the adaptive immune response and target infectious agents and damaged or altered cells. They are characterized by an increased expression of antigen-presenting molecules (e.g. MHC class II), co-stimulatory receptors for lymphocytes (e.g. CD86 and CD40), as well as a number of pro-inflammatory cytokines (e.g. IL6, IL12, IL23 and TNFα), and are reported to have microbicidal and tumoricidal activity. Alternatively activated M2 macrophages are engaged in wound healing and in the maintenances of tissue homeostasis and housecleaning and express high levels of pattern recognition receptors (e.g. Mannose receptor (MR) and scavenger receptors (e.g CD163). However, many of the functions of M2 macrophages can in fact be pro-tumorigenic since they stimulate cellular proliferation, tissue remodelling, angiogenesis and the development of an immunosuppressive environment by secretion of immune-suppressive cytokines (e.g. IL10 and TGFβ), which can be utilized by a growing tumor to invade the surrounding tissue and spread to distant organs [6]. M1 and M2 macrophages have distinct chemokine profiles, leading to the selective recruitment of immune cells, e.g. different subsets of T-lymphocytes. While M1 macrophages mainly express CXCL9 and CXCL10 which recruit lymphocytes of the T helper type 1 (Th1) and cytotoxic (Tc) subsets, M2 macrophages instead primarily recruit lymphocytes of a regulatory phenotype (Treg) and T helper type (Th2) subsets by secretion of the chemokines CCL17, CCL22 and CCL24 [4], [5], [7]. Macrophages can also be differentiated into various M2-like functional states, which have been evidenced both *in vitro* and *in vivo*, reviewed in [4]. In reality, the variations of macrophages with various M1 or M2 characteristics seem to be endless. M1 and M2 macrophage phenotypes should therefore be looked upon as extreme functional states in a spectrum of macrophage phentoypes [8]. However, the M1 and M2 classification can still be used to identify the main phenotype and functions of different macrophage populations. The macrophage phenotype is controlled by events in the tumor microenvironment in which it is found. Examples of macrophage polarizing events are secretion of tumor-derived mediators, hypoxic or necrotic factors and tissue damage [9]. The macrophage phenotype is also influenced by other immune cells and stromal components.

In human cancers, macrophage infiltration has often been correlated to a worse prognosis and therefore tumor-associated macrophages have been suggested to be mainly of an M2 phenotype [10]–[12]. However, this is not the case in all cancers. In colorectal cancer (CRC), we and others have shown that a high number of tumor-associated macrophages are correlated to an improved prognosis [13]–[17]. We have further studied the distribution of M1 and M2 macrophage phenotypes in CRC. We found that the numbers of M1 and M2 macrophages were highly correlated. Patients having high numbers of infiltrating M1 macrophages, also had high numbers of infiltrating M2 macrophages, and a significantly better prognosis [18]. Considering the high plasticity of macrophages, one possible explanation to the mixed population of M1 and M2 macrophages seen in CRC might be either that tumor secreted factors in CRC have the potential to trigger or sustain M1 macrophage features, or that they are not driving M2 macrophage differentiation to the same extent as in other cancers where macrophage infiltration is clearly detrimental for patient prognosis.

Here we have used an *in vitro* cell culture system to compare the phenotype (and functions) of tumor activated macrophages (TAMs) in CRC to that of the established M1 and M2 macrophage phenotypes to get a better understanding of how the macrophage phenotype is affected by tumor secreted factors and how this might affect patient outcome. We found that secreted factors from CRC cells did not induce a complete M1 or M2 macrophage response, but instead TAMs of a “mixed” M1/M2 phenotype. Furthermore, even though M1 and M2 macrophages were found to be easily re-edjucated in the opposite direction, secreted factors from CRC cells were unable to skew already present M1 macrophages towards M2 macrophages, but instead appeared to reinforce the M1 phenotype. Together, this might contribute to creating a “good inflammatory response” where the tumor-suppressive functions of macrophages are dominating.

## Materials and Methods

### Ethics Statement

Human monocytes were obtained from buffy coats of anonymous healthy blood donors who had given their informed consent in writing (according to local guidelines at the Blood center, Umeå University Hospital). According to the local research ethics committee (Regional Ethical Review Board in Umeå, Sweden) ethics approval is not required to study leukocytes isolated from buffy coats obtained from anonymous blood donors (dnr 2012-327-31M).

### Cell Culture

The CRC cell lines RKO, SW480 and Caco2 (ATCC) were grown in DMEM supplemented with 10% fetal bovine serum (FBS) in 37°C with 5% carbon dioxide. Conditioned media from CRC cell lines was prepared by washing cells in phosphate buffered saline (PBS), after which cells were grown to approximately 90% confluence in RPMI supplemented with 10% FBS and antibiotics for 48 hours. Conditioned media was collected, centrifuged at 3000 rpm for 30 minutes and stored in the −80°C freezer until use. Peripheral blood mononuclear cells (PBMCs) were purified from blood donor buffy coats by Dextran sedimentation and Fiqoll-Paque gradient centrifugation. Monocytes were further purified from the PBMCs either by 1,5 hours of adhesion followed by 2 washes in warm PBS supplemented with 5% FBS, or by magnetic-activated cell sorting (MACS) according to manufacturer’s instructions (Miltenyi biotec) resulting in a population of monocytes of approximately 95% purity. Purified monocytes were grown in RPMI with 10% FBS and antibiotics supplemented with 20 ng/ml M-CSF, with the medium exchanged every second day. At day 6, monocytes were further stimulated for 40 h by the addition of 100 ng/ml LPS and 20 ng/ml IFNγ (for M1 macrophages), or 20 ng/ml IL4 or IL10 (for M2 macrophages). For tumor activated macrophage subsets, monocytes were at day 6 incubated in tumor conditioned media supplemented with 20 ng/ml M-CSF for 40 h. Cells were harvested and subjected to further analyses. The differentiated macrophage phenotypes were evaluated for each buffy coat by flow cytometric analyses of expression of M1 and M2 typical markers. Cell death (apoptosis/necrosis) was controlled with flow cytometry using Annexin V/PI staining (Abcam) as recommended by the manufacturer. EtOH was added at a concentration of 5% for 30 minutes as a positive control for cell death.

For morphological investigation 8×10^6^ purified mononuclear cells per well were seeded in 6-well culture plates, further purified by adhesion, and differentiated into different macrophage populations, as described above. Live photography was taken with a DeltaPix Invenio 3S mounted on a Leitz Diavert microscope.

### Flow Cytometric Analysis

Surface marker expression was analyzed by flow cytometry (BD FACS Calibur Flow Cytometer) using R-phycoerythrin (R-PE)-conjugated monoclonal antibody against CD14 (clone M5E2, BD Pharmingen) and CD1a (clone HI149, ImmunoTools); Fluorescein isothiocyanate (FITC)-conjugated monoclonal antibody against CD86 (clone FUN-1, BD Pharmingen), HLA-DR (clone MEM12, Abcam), and CD40 (clone 5C3, BD Pharmingen); Allophycocyanin (APC)-conjugated monoclonal antibody against Mannose receptor (MR) (clone 15-2, Biolegend) and CD163 (clone 215927, R&D Systems). Matched isotype controls were included in all experiments. Before staining, Fc receptors were blocked with 5% human TruStain FCX™ (Biolegend). Data were analyzed with the CellQuest Pro software (Tree Star) after gating on the macrophage population in the FSC/SSC window.

### Endocytosis

FITC-dextran (Sigma-Aldrich) internalization was used to monitor endocytosis by macrophage populations. Monocytes were subjected to MACS purification and differentiated for 6 days in RPMI supplemented with 10% FBS and 20 ng/ml M-CSF. On day 6, macrophages were harvested and cell numbers were adjusted to 5×10^5^ cells in 1 ml of RPMI medium and seeded into a 24-well plate. The macrophages were further differentiated into the different subtypes of macrophages as described above. After 40 hours of stimulation, cells were pre-incubated on ice for 30 minutes and then incubated with 20 µg/ml FITC-dextran for 90 min at 37°C or at 4°C to detect cell surface binding. Cells were washed three times using 1 ml of PBS with 5% FBS and mean fluorescence intensities (MFIs) were determined by flow cytometry.

### Cell Migration

Boyden chamber experiments were performed to analyze how the migration of differentiated macrophages was influenced by conditioned media from CRC cells. 75 000 macrophages of different phenotypes, as indicated, were placed in a 24-well cell culture insert (8 µm pore size; BD Biosciences) in 500 µl RPMI culture medium with 10% FBS and allowed to adhere for 3 hours. Next, culture inserts were placed in conditioned medium from RKO, SW480 or Caco2 CRC cells or RPMI cell culture medium containing 10% FBS and incubated for 24 h. After wash in PBS, the inserts were placed in ice-cold methanol for 1 minute and washed again in PBS. Cells adhering to the inside of the insert were scraped off with a cotton swab, and the cells on the outside were stained with 0.5% Coomassie blue. After washes in PBS, the filter was cut out and cells were counted in three randomly selected fields and displayed as mean number of migrating cells/field.

### Cytokine and Chemokine PCR Array

The gene expression profiles of the different macrophage populations were studied with the Cytokines & Chemokines PCR Array (PAHS-150A, SABiosciences) in accordance with the manufacturer’s recommendations. Briefly, total RNA was isolated from different cell populations using The NucleoSpin® RNA II kit (Macherey-Nagel). 1 µg of total RNA was treated with DNase and cDNA was prepared using RT^2^ First Strand kit. For each analysis, cDNA samples were mixed with RT^2^ qPCR Master mix and distributed across the PCR array 96-well plates and cycled with real-time PCR (ABI 7900HT, Applied Biosystems). Amplification data (fold-changes in C_t_ values of all the genes) was analyzed with SABiosciences software.

### Statistics

Statistical significance of differences was assessed using 2-tailed students’s *t* test. A *p* value of less that 0.05 was considered statistically significant. Figures show an average of 3 independent experiments, unless otherwise stated. Error bars indicate standard deviation (SD).

## Results

To get a better understanding for how the tumor as such affect the phenotype and function of macrophages in CRC, we have used an *in vitro cell* culture model to study interactions of differentiated peripheral blood macrophages and CRC cell lines. In an *in vitro* setting, the classically activated M1 macrophage phenotype can be achieved by exposure to TLR ligands such as LPS and the Th1 typical cytokine IFNγ. Alternatively activated M2 macrophages differentiate in microenvironments rich in Th2 typical cytokines (e.g. IL4 and IL13) and are sometimes referred to as wound healing macrophages. In response to immune-suppressive cytokines (e.g. IL10) macrophages adopt an M2-like immune-regulatory phenotype [4], [8].

### Verification of M1 and M2 Macrophage Populations

**Figure 1:**
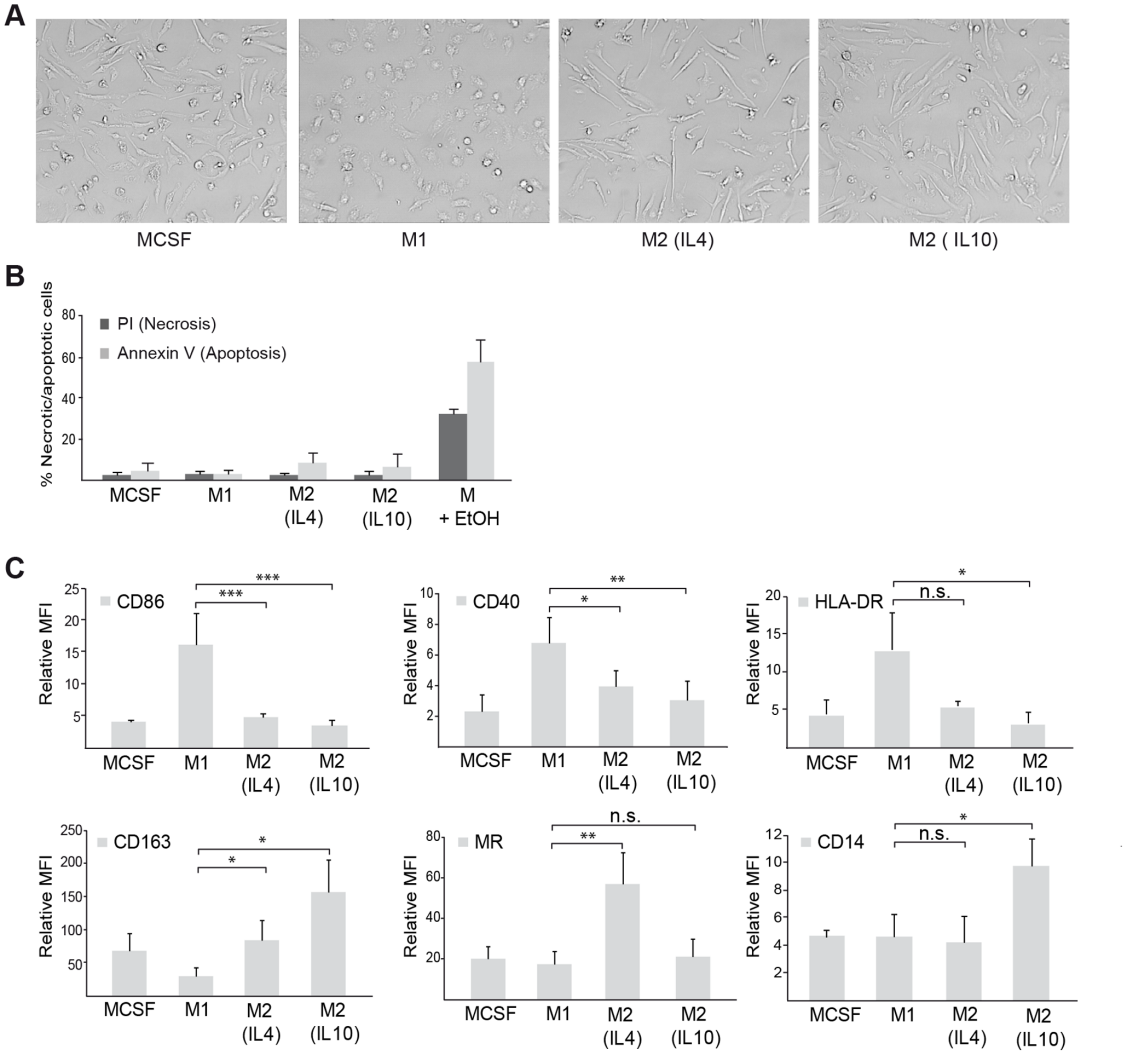
Differentiation of distinct M1 and M2 macrophage populations. (A) Morphological evaluation of macrophage phenotypes. (B) Apoptosis and necrosis of macrophage populations was evaluated by staining with Annexin V and PI, respectively, followed by flow cytometry. Shown is percent necrotic/apoptotic cells (mean ± SD) from three independent experiments. (C) Expression of extracellular markers for macrophage populations as evaluated by immunostaining and flow cytometry. Relative mean flourescense intensity (MFI) ± SD of three or more independent experiments is shown, where each sample was normalized against its respective isotype control. Relevant statistically significant differences are indicated by * (p<0.05), ** (p<0.01), or *** (p<0.001), non-significant *p* values are indicated by n.s.

Purified monocytes were differentiated to adherent macrophages by stimulation with the cytokine M-CSF for 1 week and further differentiated by the addition of LPS and IFNγ (for M1), and IL4 or IL10 (for M2 macrophages). As shown in figure 1A, M1 macrophages, in general, displayed a more round morphology while M2 macrophages were more elongated. Macrophages driven in the presence of M-CSF alone also showed an elongated morphology more resembling the M2 phenotype, even though less pronounced that that for the different populations of M2 macrophages. Looking into more detail on the morphology, IL4 stimulated M2 macrophages showed the most pronounced elongation, with especially long protrusions (Figure 1A). IL10 stimulated macrophages were more similar to the M-CSF stimulated macrophages, with the exception of being slightly more adherent. To rule out that the morphological differences seen between M1 and M2 macrophages were not due to increased apoptosis or necrosis, we analysed the binding of Annexin V to cell surface phosphatidylserine and as well as propidium iodine (PI) uptake, respectively. As shown in figure 1B, the different macrophage phenotypes contained reasonable low levels of apoptotic and necrotic cells, respectively. When looking at the expression of M1 or M2 typical markers by flow cytometry, M1 macrophages were shown to express high cell surface levels of the MHC class II receptor HLA-DR, as well as T cell co-stimulatory receptors, CD86 and CD40, but lower levels of the typical M2 markers, CD163 and MR (Figure 1C). M2 macrophages instead expressed high levels of CD163 and MR, while showing low expression of the M1 markers (Figure 1C). The two M2 populations could further be distinguished by that the IL4 stimulated macrophages showed higher expression of MR, while IL10 stimulated macrophages expressed higher levels of CD163 (Figure 1C). Furthermore, all macrophage subtypes were found to typically express the monocyte/macrophage marker CD14 (Figure 1C). CD1a, the marker for the dendritic cells of monocytic origin, was not expressed by either of the macrophage populations (data not shown).

### Migration of M1 and M2 Macrophages

**Figure 2:**
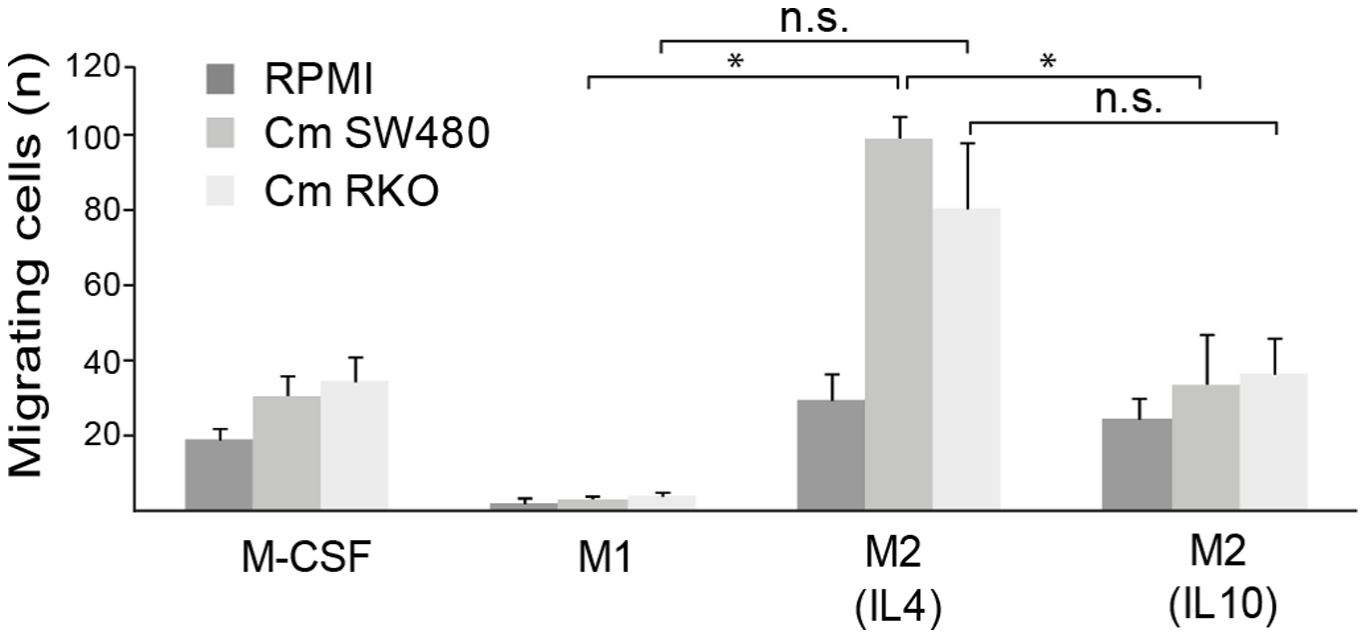
The migratory ability of M1 and M2 macrophage populations. Migration of macrophage phenotypes was evaluated by Boyden chamber experiments, where macrophages were allowed to migrate towards conditioned media from RKO or SW480 CRC cell lines, or culture medium control (RPMI+10% FBS). Mean numbers of migrating cells (n) ± SD is shown. Relevant statistically significant differences are indicated by * (p<0.05), non-significant *p* values are indicated by n.s.

To study whether tumor secreted factors in some way preferentially recruit macrophages of a certain phenotype, we looked at the migratory ability of M1 and M2 macrophages in an *in vitro* cell migration assay. We found that M1 macrophages had very low migratory ability towards RPMI control medium, while M-CSF stimulated and M2 macrophages migrated more towards medium alone (Figure 2). When allowing the different subpopulations of macrophages to migrate towards conditioned media from RKO or SW480 CRC cell lines, we found that preferentially IL4 stimulated M2 macrophages showed increased migration towards tumor conditioned media, suggesting that tumor secreted factors preferably recruit macrophages of a wound-healing phenotype (Figure 2).

### The Phenotype of Tumor Activated Macrophages (TAMs)

**Figure 3:**
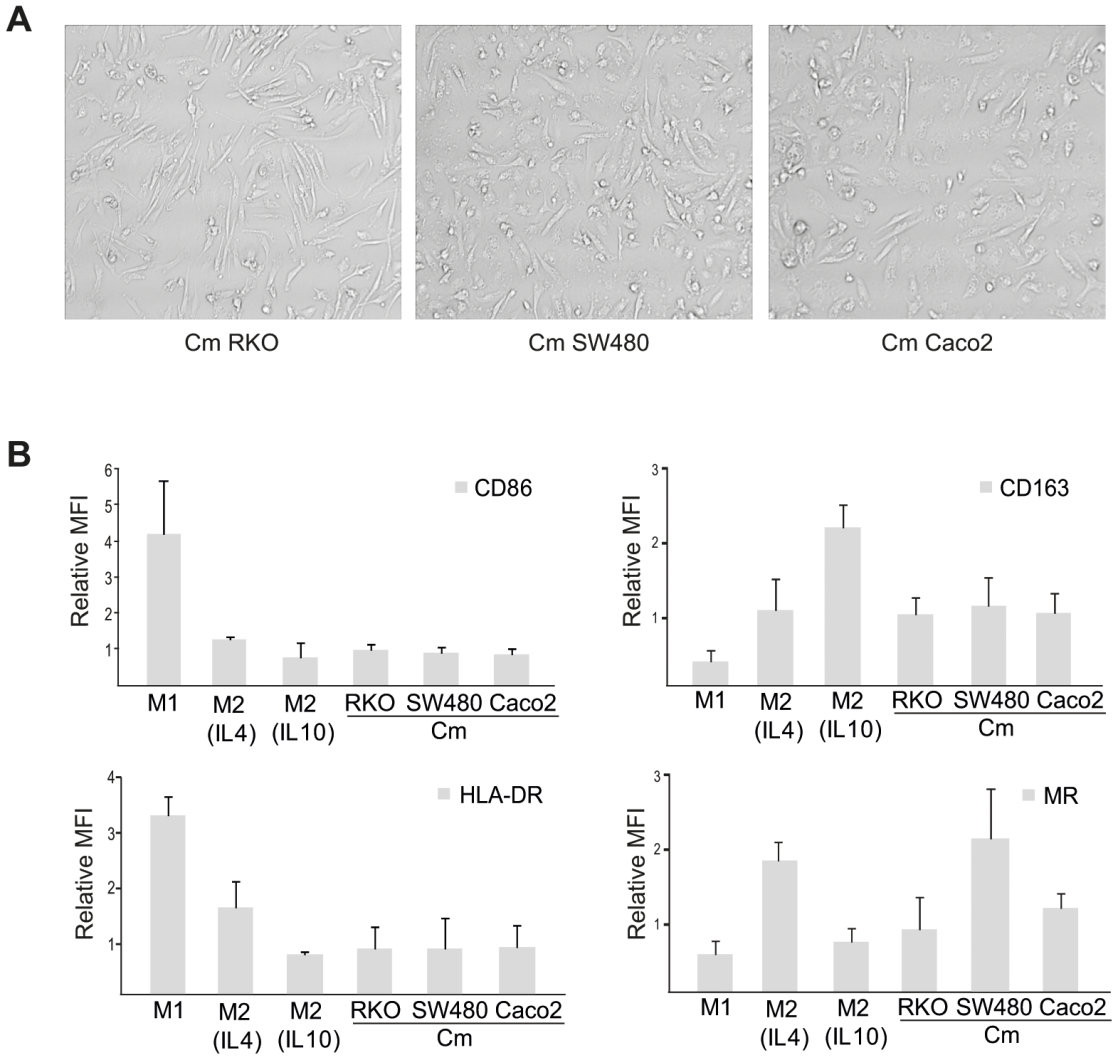
Effects on the macrophage phenotype by secreted factors from CRC cells. (A) Morphological evaluation of macrophages stimulated with conditioned media (Cm) from RKO, SW480 or Caco2 cell lines. (B) Expression of extracellular markers was evaluated by immunostaining and flow cytometry in macrophages of M1 and M2 subtype or in macrophages stimulated with conditioned media (Cm) from CRC cell lines. Relative mean flourescense intensity (MFI) ± SD of three or more independent experiments is shown, where each sample was normalized against its respective isotype control and with the M-CSF stimulated control sample set as 1.

The morphology and cell surface phenotype of TAMs differentiated in the presence of conditioned media from RKO, SW480 or Caco2 CRC cell lines was analysed in figure 3A and B, respectively. TAMs were elongated, thus the morphology more resembled that of M2 macrophage populations (Compare Figures 3A and 1A). When looking at the expression of M1 and M2 specific cell surface markers, TAMs were also found to share more similarities with M2 macrophages (Figure 3B). However, the TAM phenotype was not as pronounced as that for the M2 macrophages, since the high expression of either MR (as in IL4 stimulated M2 macrophages) or CD163 (as in IL10 stimulated M2 macrophages) was not generally seen. The exception was macrophages activated by conditioned media from SW480 cells, which displayed a high expression of MR similar to that of IL4 stimulated M2 macrophages (Figure 3B). These data therefore suggest that SW480 cells can skew macrophages towards a pronounced M2 phenotype, while RKO or Caco2 cells does not induce major changes to the M-CSF stimulated macrophages, at least not changes that were detected here.

### Endocytosis Capacity of M1/M2 Macrophage Populations and TAMs

**Figure 4:**
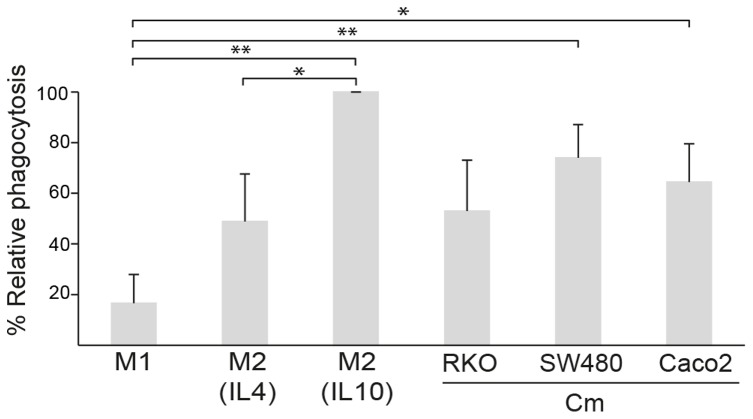
The endocytic ability of the different macrophage populations. Endocytosis by M1 and M2 macrophages, or macrophages stimulated with conditioned media (Cm) from RKO, SW480 or Caco2 CRC cell lines was evaluated by measuring internalization of FITC-dextran by flow cytometry. Shown is percent relative endocytosis (mean ± SD) with endocytosis by IL10 stimulated M2 macrophages set as 100%. Statistically significant differences are indicated by * (p<0.05) or ** (p<0.01). All other differences were tested but found non-significant (not indicated in figure).

The endocytic function of the different subsets of M1 and M2 macrophages, as well as TAMs, was also investigated in our *in vitro* cell culture system. Cells were differentiated into the different macrophage phenotypes, and monitored for their ability to endocytose fluorescently labelled dextran. M1 macrophages were shown to have a lower endocytic ability compared to M2 macrophages, approximately 20% for M1 compared to 50–100% for M2 macrophages, with IL10 stimulated M2 macrophages displaying the highest endocytic ability (Figure 4). In order to investigate how tumor secreted factors affect macrophage endocytosis, we compared that of M1, M2 and TAMs (Figure 4). This analysis showed that endocytosis of FITC-dextran of TAMs more resembled that of M2 macrophages.

### Macrophage Plasticity

**Figure 5:**
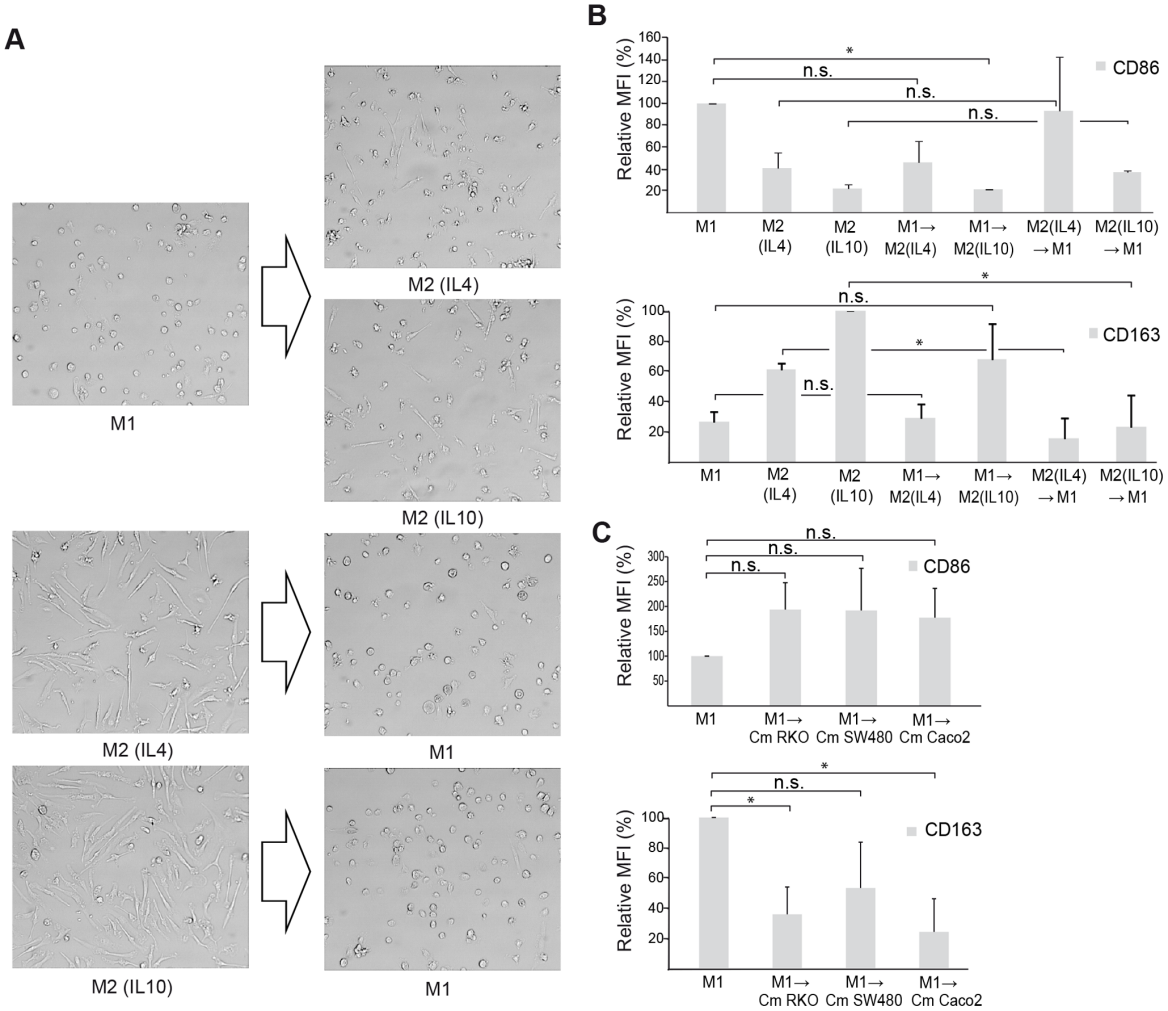
Macrophages are plastic cells able to switch between different phenotypes. The morphology and expression of extracellular markers was evaluated in macrophages of distinct M1 or M2 subtypes before and after stimulation towards the opposite phenotype or stimulation by conditioned media (Cm) from RKO, SW480 or Caco2 CRC cell lines. (A) Morphological evaluation of macrophage phenotypes. (B) Expression of extracellular markers for macrophage populations as evaluated by immunostaining and flow cytometry. (C) Differentiation of M1 macrophages by stimulation with conditioned media (Cm) from CRC cell lines. Relative mean flourescense intensity (MFI) ± SD of three independent experiments is shown, where each sample was normalized against its respective isotype control and with the M1 or M2 (IL10) stimulated macrophage sample set as 100%. Relevant statistically significant differences are indicated by * (p<0.05), non-significant *p* values are indicated by n.s.

Macrophages are known to be plastic cells, adopting various shapes depending on factors in the milieu in which they are found. When analysing plasticity in our *in vitro* cell culture system, the M1 and M2 macrophages were indeed found to be very plastic in nature, since differentiated M1 macrophages could be driven towards a M2 phenotype, and vice versa, as evaluated by morphological investigation and expression of M1- and M2-typical macrophage markers (Figure 5). The morphology of M1 macrophages that received M2 stimuli was shown to switch to a more elongated M2 appearance (Figure 5A), even though not completely. On the other hand M2 macrophages appeared to more quickly adopt the round morphology of M1 macrophages when subjected to the opposite stimuli (Figure 5A). When analysing expression of M1 and M2 markers, a shift between phenotypes was also evident, since M1 typical markers were found to be down-regulated by M2-stimuli and vice versa (Figure 5B). It is suggested in the literature that TAMs are skewed by tumor secreted factors towards an M2 phenotype [10]–[12]. However, this was not clearly reflected in our *in vitro* cell culture system, since a distinct M2 population was not generally induced by secreted factors from CRC cells. Because of the plastic behaviour displayed by the macrophage populations, we sought to investigate if differentiated M1 macrophages could be affected by tumor secreted factors. However, conditioned media from CRC cells was not able to skew already differentiated M1 macrophages towards an M2 phenotype. Instead, conditioned media from CRC cells increased the expression of the M1 marker CD86, while reducing that of M2 marker CD163, and thereby appeared to reinforce the M1 phenotype (Figure 5C). IL4 or IL10 stimulated M2 macrophages were not affected by secreted factors from CRC cells (data not shown). This suggests that tumor secreted factors alone are not able to shift an established M1 macrophage phenotype towards an M2 phenotype in CRC.

### Cytokine Expression by M1 and M2 Macrophages and TAM Populations

**Figure 6:**
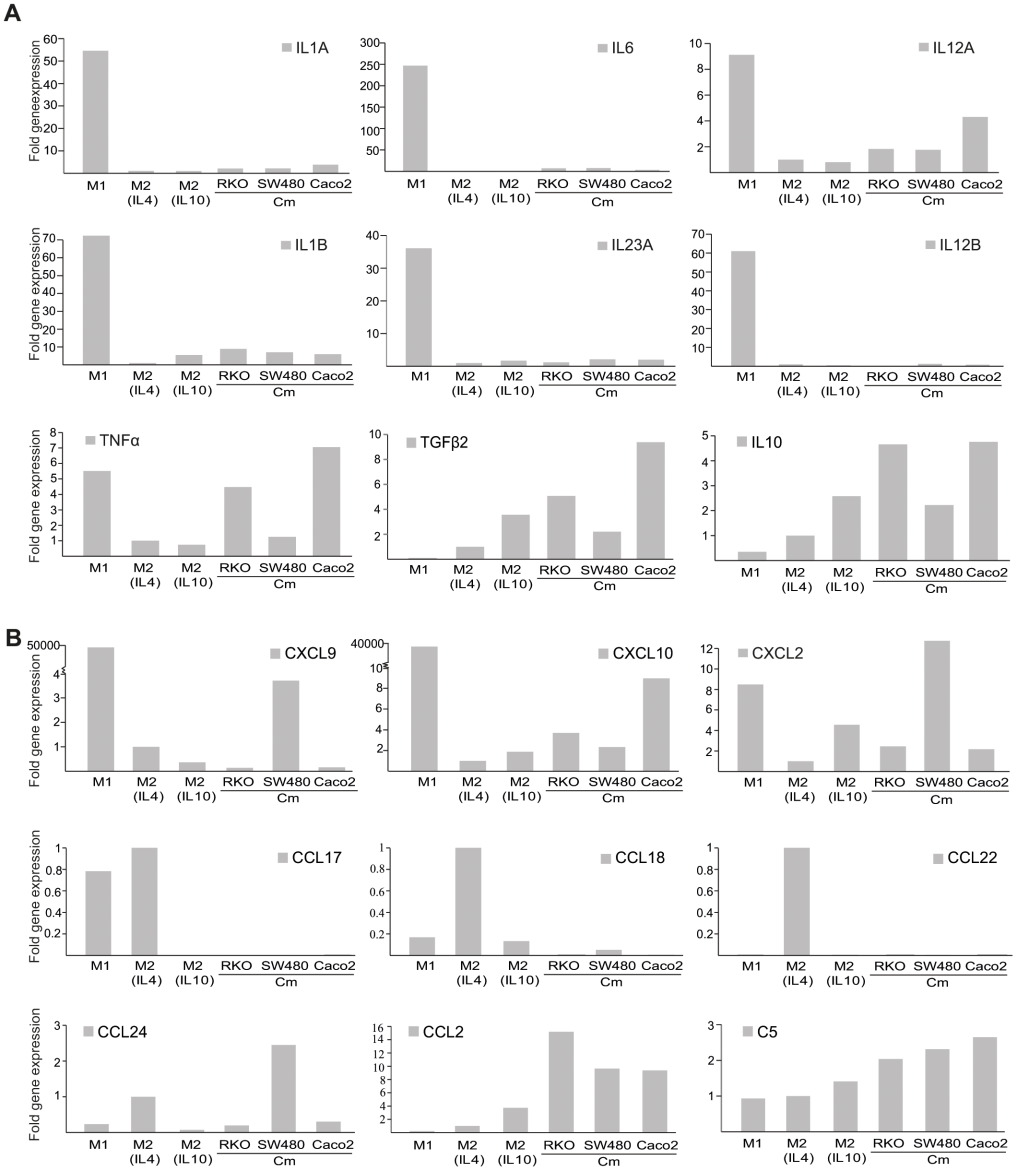
The gene expression profiles of the different macrophage populations. Gene expression was studied in M1 and M2 macrophages, or macrophages stimulated with conditioned media (Cm) from RKO, SW480 or Caco2 CRC cell lines using a Cytokine & Chemokine PCR Array. (A) Expression of pro-inflammatory and anti-inflammatory cytokines. (B) Expression of chemotactic factors. Shown is fold gene expression, with IL4 stimulated M2 macrophages set as 1.

It is apparent that the simplified distinct definition of M1 and M2 macrophages does not apply to all macrophages in an *in vivo* setting. However to analyse the differences that are caused by tumor secreted factors alone, we again utilized our *in vitro* cell culture system to look at potential differences and similarities between the cytokine and chemokine expression patterns of M1 and M2 macrophages, or TAM populations. For this, we used a PCR-based cytokine and chemokine gene expression array (results presented in detail in table S1). Selected genes of interest are presented in figure 6. As expected, M1 macrophages were found to express higher levels of the pro-inflammatory cytokines, IL1, IL6, IL12, IL23 and TNFα, which were not expressed by the M2 macrophages (Figure 6A). The immune regulatory cytokines IL10 and TGFβ were mainly expressed by M2 macrophages, in particular IL10-derived M2-like immune-regulatory macrophages (Figure 6A). When looking at the cytokine and chemokine expression pattern in TAMs it was clear that each CRC cell line induced an individual expression pattern. When comparing the gene expression profile of M1 and M2 macrophages to that of the different TAMs, TAMs were found to express higher levels of pro-inflammatory cytokines than M2 macrophages, even though it in most cases was much lower than for the M1 macrophages. One exception was TNFα, which was found to be expressed by TAMs to similar levels as for M1 macrophages. The immune-regulatory chemokines IL10 and TGFβ2 were expressed also by the different TAMs, in some cases to an even higher extent than the M2 macrophages. We sought to investigate the differences in the chemotactic response induced by M1 or M2 macrophages and TAMs to understand how the tumor in CRC affects the macrophage induced recruitment of other immune cells (Figure 6B). When looking at factors that preferentially recruits lymphocytes of the Th1 type (e.g. CXCL9 and CXCL10), TAMs stimulated by conditioned media from RKO or Caco2 cells were found to express higher levels than M2 macrophages, but still very low amounts as compared to the expression of these factors by M1 macrophages. The chemotactic factor for neutrophils CXCL2 [19] was secreted by TAMs stimulated by conditioned media from SW480 cells. This suggests that, although the effects were modest, some features of M1 macrophages can be found also in TAMs. When looking at factors that recruit lymphocytes of the Treg and Th2 type (e.g. CCL17, CCL18, CCL22 and CCL24), which are expressed mainly by the M2 population, these were found to not be expressed by TAMs, with the exception of CCL24 in TAMs stimulated by conditioned media from SW480 cells. This suggests that not all M2 features are found in TAMs. Finally, we looked at genes that were more highly expressed in TAMs in general, compared to that in M1 or M2 macrophages. The monocyte attracting chemotactic factor CCL2 was found to be highly expressed in all TAM populations. Also complement protein C5 was found to be highly expressed in TAMs in particular. Together, this suggest that tumor secreted factors induces TAMs of a “mixed” phenotype, with some features that are TAM specific and some features which can be similar or different to that in M1 or M2 macrophages.

## Discussion

In a previous study, where we analyzed M1 and M2 macrophage distribution in a patient cohort of CRC, we found that an increased infiltration of macrophages of an M1 phenotype could be correlated to an improved survival in CRC patients [18]. However, infiltration of M1 macrophages was found to also be accompanied by infiltration of M2 macrophages. Tumor cells are known to produce factors that affect the macrophage phenotype and thereby also the macrophage response towards the tumor, suggested to favor tumor invasion and metastasis [12], [20]. Possible explanations to the positive role of macrophages seen on prognosis in CRC, could be either that CRC somehow sustains an ongoing M1 macrophage response, or less efficiently skew the macrophage response towards an M2 response than other cancers where infiltration of macrophages is a bad prognostic marker, thus creating TAMs with less hazardous tumor promoting effects.

Here, we have used an *in vitro* cell culture system to analyze if secreted factors from CRC cells can affect the macrophage phenotype. We have compared the phenotype (morphology, antigen-presentation, migration, endocytosis, and expression of cytokine and chemokine genes) of M1, M2 and TAMs. We found that secreted factors from CRC cells alone were unable to induce a strong M1 macrophage phenotype as well as, at least in most cases, a pronounced M2 wound-healing or M2-like immunosuppressive phenotype. The lack of most M1 features in TAMs, suggests that either the M1 differentiation is dependent on direct interactions between macrophages and tumor cells, or that there are other microenvironmental stimuli required for the development of M1 macrophages in CRC. However, direct cellular interactions are hard to study in human cell culture models because of the problems with self to non-self recognition. It might also be that it is not the mutated tumor cell itself that triggers the M1 response. Solid tumors of the colorectum represent an example of a clearly dysregulated tissue, that could affect the macrophage response by many different means [9]. The M1 response might thus be provoked by other influences, such as bacterial infiltration or other changes in the tumor microenvironment (e.g. hypoxia, necrosis or fibrosis) that is not reflected in our *in vitro* system. Interaction with other cells of tumor stroma, as well as other immune cells, might also be required for M1 macrophage development. Indeed, a high infiltration of one type of immune cells has been correlated to increased infiltration of also other types of immune cells, and to a general inflammatory reaction along the tumor invasive margin in colorectal cancer [21]–[23]. How the net immune reaction is composed is likely to influence the macrophage response in different ways. These are interesting questions that needs to be further addressed. Since macrophages are highly plastic cells, macrophage populations functionally and reversibly adapt to changes in the environment, which has been previously documented [24], [25]. We could demonstrate that differentiation of M1 and M2 macrophages was reversible and could be driven in the opposite direction by counterstimulatory cytokines. This might have important therapeutic advantages in the treatment of chronic disease such as cancer, were therapeutic interventions could be used to manipulate the macrophage response towards an anti-tumorigenic response in favour of patient survival. The reversibility of the functional adaptation of macrophages suggested that these cells are not terminally differentiated and thereby also differentiated M1 and M2 macrophages could be affected by tumor secreted factors. We found that M1 macrophages in CRC were not skewed towards M2 macrophages by tumor secreted factors. Instead these factors slightly enhanced the M1 response. M1 macrophages had very little ability to migrate and low endocytic ability, while the wound-healing IL4 stimulated M2 macrophages, as well as the immune-regulatory IL10 stimulated M2-like macrophages, instead had a high migratory and endocytic capacity. TAMs were found to share more similarities with M2 macrophages. However, based on morphology and marker expression, secreted factors from CRC cells were in most cases unable to induce a complete switch towards M2 macrophages. To search for additional M1 or M2 macrophage characteristics in TAMs we compared the cytokine and chemokine expression patterns of M1 and M2 macrophages, as well as TAMs. This comparison revealed that TAMs appear to be of a “mixed” M1/M2 macrophage phenotype. We found that TAMs share expression of pro-inflammatory cytokines, in particular TNFα and IL12, with M1 macrophages. TNFα is an important activator of acute inflammation that appears to play a complex and dual role in cancer, but has at higher doses been shown to have anti-tumor activity [26]. IL12 plays an important role in the activation of natural killer cells and T lymphocytes, stimulating their production of IFNγ, which favours the differentiation of Th1 lymphocytes thereby connecting innate and adaptive immunity [27]. TAMs also share the expression of some chemotactic factors for Th1 and Tc lymphocytes as well as neutrophils (e.g. CXCL9, CXCL10 and CXCL2). In addition, TAMs were found to lack expression of some of the chemotactic factors that are expressed by M2 macrophages, which recruit lymphocytes of the Th2 and Treg subsets thereby being part of creating an immune-supressive environment. It might be that these subtle differences between TAMs in CRC and the strict M2 macrophage phenotypes could contribute to the maintenance of a “good inflammatory response” and the positive role of macrophages seen in CRC patient prognosis [28]. If this is enough to make the difference is of course hard to predict at this point. A pro-inflammatory TAM phenotype has been previously suggested by Ong et al [29]. In that study they used a multi-cellular tumor spheroid model, and thereby also direct cellular interactions, of a CRC cell line and peripheral blood monocytes to establish TAMs. To better understand the different roles of macrophages in cancer, further studies are required where the phenotype of TAMs in tumors of CRC patients can be compared to that of other cancers, to identify how the TAM phenotype is affected by tumor specific factors or other tumor microenvironmental traits. In fact, several adaptations of immune cells have been found in the intestine to cope with the reactive environment where immune “triggers” are continuously found [30]. In our *in vitro* cell culture system we analysed the effect of secreted factors from CRC cells on the phenotype of M-CSF stimulated macrophages, resembling resident macrophages in an *in vivo* setting. It remains however to be elucidated how tumor secreted factors would affect the differentiation of monocytes to mature macrophages, which could mirror the phenotype of newly recruited macrophages.

In conclusion, tumor secreted factors alone appear to create a mixed TAM phenotype, that share some features of both M1 macrophages or M2 macrophages, while some are typical for the TAMs. Even though *in situ* evaluation of immunohistochemical sections of CRC showed that M1 macrophages are present at the tumor front, tumor secreted factors alone were unable to trigger a M1 macrophage phenotype, but instead induced a TAM phenotype that more resembled M2 macrophages. However, it is clear from our studies, that tumor secreted factors by themselves are unable to “shift” a developed M1 macrophage phenotype towards an M2 macrophage phenotype, suggesting that this shift is caused by the recruitment of other cells of the immune system or by other changes in the tumor microenvironment. The finding that TAMs express some pro-inflammatory cytokines, as well as chemokines affecting the recruitment of inflammatory neutrophils and Tc and Th1 lymphocytes, while lacking expression of M2 macrophage related Th2 and Treg recruiting chemokines, might be part of an explanation to the positive role seen by macrophages in CRC. Further studies are required to understand how the phenotype of macrophages is changing in the tumor microenvironment and subsequently how this affects tumor growth and spread. This might contribute to important therapeutic advances, where tumor associated macrophages can be re-edjucated to a tumor-suppressive phenotype and elicit a potent anti-tumor immune response.

## Supporting Information

Table S1 **Full list of gene expression array data.**
(DOCX)
