# Supplementary material for: Phenotypic Skewing of Macrophages In Vitro by Secreted Factors from Colorectal Cancer Cells
Source: PLoS One. 2013 Sep 18;8(9):e74982. doi: 10.1371/journal.pone.0074982 (PMC3776729; doi:10.1371/journal.pone.0074982)
Supplement: Table S1 — Full list of gene expression array data. (DOCX) [file pone.0074982.s001.docx]

**Table S1. Full list of gene expression array data.**

| **Gene symbol** | **M1** | **M2 (IL4)** | **M2 (IL10)** | **M (Cm RKO)** | **M (Cm Sw480)** | **M (Cm Caco2)** |
| --- | --- | --- | --- | --- | --- | --- |
| **ADIPOQ** | 2,01E-05 | 7,11E-05 | 1,60E-05 | 3,54E-06 | 7,16E-06 | 9,15E-06 |
| **BMP2** | 5,81E-04 | 3,92E-05 | 4,27E-05 | 2,29E-05 | 4,35E-05 | 1,94E-04 |
| **BMP4** | 3,43E-05 | 4,01E-05 | 4,70E-05 | 6,57E-05 | 4,48E-05 | 8,31E-05 |
| **BMP6** | 1,11E-03 | 4,04E-04 | 1,39E-04 | 2,17E-04 | 1,16E-04 | 2,69E-04 |
| **BMP7** | 1,30E-04 | 1,21E-05 | 4,69E-06 | 3,54E-06 | 5,63E-06 | 3,62E-06 |
| **C5** | 5,59E-03 | 6,00E-03 | 8,45E-03 | 1,22E-02 | 1,39E-02 | 1,59E-02 |
| **CCL1** | 3,31E-04 | 8,16E-04 | 1,09E-04 | 1,06E-03 | 2,58E-03 | 3,05E-04 |
| **CCL11** | 7,91E-06 | 3,86E-05 | 7,71E-06 | 4,19E-06 | 4,08E-06 | 1,26E-05 |
| **CCL13** | 2,82E-04 | 5,39E-01 | 1,57E-03 | 1,42E-03 | 3,12E-01 | 9,68E-04 |
| **CCL17** | 2,90E-03 | 3,71E-03 | 7,80E-06 | 3,54E-06 | 1,41E-05 | 2,90E-05 |
| **CCL18** | 7,49E-02 | 4,44E-01 | 5,91E-02 | 4,06E-03 | 2,26E-02 | 2,37E-03 |
| **CCL19** | 1,21E+00 | 9,26E-06 | 2,04E-05 | 5,60E-06 | 1,01E-05 | 1,56E-05 |
| **CCL2** | 6,25E-02 | 2,73E-01 | 1,02E+00 | 4,15E+00 | 2,64E+00 | 2,56E+00 |
| **CCL20** | 4,27E-03 | 2,00E-05 | 9,14E-05 | 6,09E-05 | 9,78E-06 | 1,75E-04 |
| **CCL21** | 1,11E-04 | 2,10E-06 | 4,69E-06 | 3,54E-06 | 4,08E-06 | 3,62E-06 |
| **CCL22** | 4,42E-03 | 5,37E-01 | 3,24E-03 | 5,14E-03 | 2,75E-03 | 6,01E-03 |
| **CCL24** | 1,63E-04 | 7,03E-04 | 5,11E-05 | 1,37E-04 | 1,72E-03 | 2,13E-04 |
| **CCL3** | 3,51E-01 | 7,60E-02 | 1,60E-02 | 2,49E-03 | 4,73E-02 | 9,65E-03 |
| **CCL5** | 1,99E+00 | 1,05E-02 | 2,90E-03 | 1,79E-03 | 4,49E-03 | 1,96E-03 |
| **CCL7** | 7,24E-04 | 8,98E-02 | 1,41E-02 | 3,44E-02 | 3,14E-01 | 1,01E-02 |
| **CCL8** | 7,03E-01 | 3,29E-01 | 5,43E-02 | 1,67E-02 | 2,21E-01 | 1,67E-02 |
| **CD40LG** | 8,91E-04 | 2,13E-05 | 1,00E-04 | 1,27E-04 | 1,51E-04 | 3,14E-04 |
| **CNTF** | 4,09E-03 | 7,88E-04 | 1,69E-03 | 7,28E-04 | 1,49E-03 | 3,28E-03 |
| **CSF1** | 3,09E-02 | 1,16E-02 | 5,75E-03 | 7,77E-04 | 2,74E-02 | 5,00E-03 |
| **CSF2** | 5,18E-06 | 2,10E-06 | 4,69E-06 | 3,54E-06 | 2,69E-05 | 3,62E-06 |
| **CSF3** | 3,06E-03 | 2,10E-06 | 4,69E-06 | 3,54E-06 | 6,91E-06 | 4,30E-06 |
| **CX3CL1** | 2,16E-05 | 2,06E-05 | 2,05E-05 | 3,69E-05 | 1,90E-05 | 4,21E-05 |
| **CXCL1** | 3,96E-02 | 1,77E-02 | 6,07E-02 | 1,26E-02 | 9,63E-02 | 2,45E-02 |
| **CXCL10** | 1,76E+01 | 4,77E-04 | 8,94E-04 | 1,76E-03 | 1,11E-03 | 4,27E-03 |
| **CXCL11** | 6,17E+00 | 5,87E-05 | 6,58E-05 | 5,05E-05 | 1,07E-04 | 3,29E-05 |
| **CXCL12** | 4,93E-03 | 2,09E-05 | 1,38E-03 | 3,58E-04 | 6,99E-05 | 3,37E-04 |
| **CXCL13** | 3,72E-01 | 1,94E-05 | 3,05E-05 | 3,54E-06 | 2,22E-05 | 1,97E-05 |
| **CXCL16** | 3,39E-01 | 4,54E-02 | 6,81E-02 | 6,67E-03 | 4,79E-02 | 7,35E-03 |
| **CXCL2** | 6,60E-02 | 7,79E-03 | 3,54E-02 | 1,91E-02 | 9,92E-02 | 1,69E-02 |
| **CXCL5** | 3,28E-02 | 6,43E-03 | 1,70E-01 | 1,08E-02 | 8,16E-01 | 5,24E-03 |
| **CXCL9** | 2,13E+01 | 4,38E-04 | 1,60E-04 | 6,25E-05 | 1,62E-03 | 7,10E-05 |
| **FASLG** | 5,92E-05 | 5,58E-06 | 4,69E-06 | 3,54E-06 | 4,08E-06 | 2,05E-05 |
| **GPI** | 6,45E-02 | 1,77E-01 | 1,54E-01 | 2,40E-01 | 1,50E-01 | 2,47E-01 |
| **IFNA2** | 8,61E-06 | 3,13E-06 | 4,69E-06 | 3,54E-06 | 4,50E-06 | 3,62E-06 |
| **IFNG** | 2,72E-05 | 2,57E-05 | 4,69E-06 | 3,54E-06 | 4,08E-06 | 4,55E-05 |
| **IL10** | 2,14E-03 | 6,22E-03 | 1,61E-02 | 2,90E-02 | 1,38E-02 | 2,96E-02 |
| **IL11** | 8,82E-06 | 2,10E-06 | 4,69E-06 | 3,54E-06 | 6,92E-06 | 8,13E-06 |
| **IL12A** | 1,70E-04 | 1,86E-05 | 1,51E-05 | 3,39E-05 | 3,29E-05 | 8,02E-05 |
| **IL12B** | 1,10E-03 | 1,80E-05 | 9,50E-06 | 3,56E-06 | 2,29E-05 | 1,42E-05 |
| **IL13** | 2,18E-05 | 2,10E-06 | 4,69E-06 | 1,48E-05 | 4,08E-06 | 2,36E-05 |
| **IL15** | 9,93E-02 | 1,75E-03 | 3,94E-03 | 3,20E-03 | 3,41E-03 | 3,79E-03 |
| **IL16** | 4,57E-04 | 2,94E-03 | 6,14E-03 | 2,59E-03 | 5,83E-03 | 7,78E-03 |
| **IL17A** | 3,41E-06 | N.A. | 4,69E-06 | 3,54E-06 | 4,08E-06 | 3,62E-06 |
| **IL17F** | 8,66E-06 | 2,24E-06 | 4,69E-06 | 3,54E-06 | 4,73E-06 | 1,54E-05 |
| **IL18** | 2,50E-01 | 3,62E-02 | 1,21E-01 | 1,26E-01 | 1,13E-01 | 1,39E-01 |
| **IL1A** | 1,87E-02 | 3,42E-04 | 3,21E-04 | 7,21E-04 | 7,58E-04 | 1,31E-03 |
| **IL1B** | 1,64E-01 | 2,26E-03 | 1,24E-02 | 2,01E-02 | 1,59E-02 | 1,33E-02 |
| **IL1RN** | 1,16E+00 | 4,36E-01 | 5,66E-02 | 5,11E-02 | 9,89E-02 | 4,67E-02 |
| **IL2** | 3,71E-05 | 2,10E-06 | 4,69E-06 | 1,09E-05 | 4,08E-06 | 1,45E-05 |
| **IL21** | 1,23E-05 | 2,39E-05 | 1,24E-05 | 3,54E-06 | 6,28E-06 | 1,08E-05 |
| **IL22** | 6,02E-05 | 1,34E-05 | 4,69E-06 | 3,54E-06 | 2,57E-05 | 1,23E-05 |
| **IL23A** | 2,27E-02 | 6,29E-04 | 1,09E-03 | 7,39E-04 | 1,34E-03 | 1,26E-03 |
| **IL24** | 9,32E-05 | 3,81E-04 | 8,30E-04 | 2,98E-04 | 7,60E-04 | 3,69E-04 |
| **IL27** | 3,80E-02 | 1,00E-04 | 1,06E-04 | 3,64E-05 | 1,04E-04 | 2,33E-04 |
| **IL3** | 1,32E-04 | 7,99E-05 | 2,01E-05 | 1,51E-05 | 8,77E-05 | 6,74E-05 |
| **IL4** | 3,41E-06 | 2,10E-06 | 1,08E-05 | 3,54E-06 | 9,34E-06 | 3,62E-06 |
| **IL5** | 1,56E-04 | 8,88E-05 | 1,45E-05 | 4,15E-05 | 7,27E-05 | 1,06E-04 |
| **IL6** | 1,21E-01 | 4,92E-04 | 4,15E-04 | 3,49E-03 | 3,72E-03 | 1,46E-03 |
| **IL7** | 2,97E-01 | 4,21E-04 | 3,14E-03 | 8,30E-04 | 1,46E-03 | 1,13E-03 |
| **IL8** | 1,48E+00 | 1,03E-02 | 3,32E-02 | 1,47E-02 | 8,36E-02 | 1,95E-02 |
| **IL9** | 9,75E-06 | 1,86E-04 | 4,69E-06 | 1,94E-05 | 7,53E-05 | 1,83E-05 |
| **LIF** | 5,48E-03 | 2,88E-04 | 3,28E-04 | 3,01E-04 | 3,29E-04 | 1,73E-04 |
| **LTA** | 9,11E-04 | 1,98E-05 | 4,69E-06 | 4,71E-05 | 6,43E-05 | 6,28E-05 |
| **LTB** | 7,36E-04 | 5,09E-04 | 4,12E-04 | 1,96E-03 | 6,72E-04 | 1,73E-03 |
| **MIF** | 7,92E-02 | 2,59E-01 | 2,43E-01 | 2,88E-01 | 2,27E-01 | 2,56E-01 |
| **MSTN** | 1,20E-04 | 9,84E-05 | 5,19E-05 | 1,43E-05 | 8,36E-05 | 1,06E-04 |
| **NODAL** | 6,95E-04 | 2,10E-06 | 1,05E-04 | 3,79E-05 | 8,57E-05 | 2,32E-04 |
| **OSM** | 6,14E-02 | 3,98E-04 | 1,60E-03 | 7,59E-04 | 2,49E-03 | 3,02E-03 |
| **PPBP** | 3,67E-04 | 3,86E-02 | 7,38E-01 | 3,35E-01 | 8,47E+00 | 2,40E-02 |
| **SPP1** | 6,17E-02 | 7,74E+00 | 2,66E+01 | 1,19E+01 | 1,96E+01 | 1,62E+01 |
| **TGFB2** | 3,77E-05 | 3,95E-04 | 1,41E-03 | 2,00E-03 | 8,73E-04 | 3,70E-03 |
| **THPO** | 2,24E-05 | 2,05E-05 | 1,44E-05 | 3,54E-06 | 5,28E-06 | 4,72E-05 |
| **TNF** | 2,50E-02 | 4,53E-03 | 3,36E-03 | 2,03E-02 | 5,67E-03 | 3,20E-02 |
| **TNFRSF11B** | 3,41E-06 | 2,77E-05 | 3,70E-05 | 3,54E-06 | 4,08E-06 | 7,85E-06 |
| **TNFSF10** | 1,23E+00 | 4,83E-03 | 3,35E-02 | 1,31E-02 | 1,54E-02 | 1,07E-02 |
| **TNFSF11** | 2,61E-04 | 4,42E-05 | 6,39E-05 | 1,33E-04 | 9,66E-05 | 1,59E-04 |
| **TNFSF13B** | 7,74E-02 | 6,22E-03 | 1,87E-02 | 1,76E-02 | 1,34E-02 | 2,80E-02 |
| **VEGFA** | 3,86E-01 | 1,72E-03 | 6,01E-03 | 3,06E-03 | 3,79E-03 | 5,85E-03 |
| **XCL1** | 3,41E-06 | 2,10E-06 | 8,06E-06 | 6,08E-06 | 1,08E-05 | 1,14E-05 |

Normalized expression of cytokine and chemokine genes, according to the 2^ -ΔCt (Ct(GOI) - Ave Ct (HKG)) data analysis method. GOI; Gene of interest, HKG; House keeping gene.
